# Characteristics of the Washington Group Short Set in Assessing Functional Difficulty Among People With Physical Disability Certificates of Vision, Hearing, and Lower-Limb Impairments in Japan

**DOI:** 10.7759/cureus.96999

**Published:** 2025-11-16

**Authors:** Takashi Saito, Kumiko Imahashi

**Affiliations:** 1 Department of Social Rehabilitation, Research Institute of National Rehabilitation Center for Persons With Disabilities, Tokorozawa, JPN

**Keywords:** disability, disability certificate, disability statistics, japan, physical disability certificate, washington group short set questionnaire on functioning

## Abstract

Introduction: The Washington Group Short Set on Functioning (WG-SS), consisting of six questions on functional difficulties, is an international disability measure aiming to estimate disability prevalence and disability-related inequalities globally. In 2022, the WG-SS was introduced for the first time in Japan, necessitating a better understanding of the characteristics of the new measure compared with an existing classic disability indicator in Japan, i.e., possession of a physical disability certificate (PDC). Considering that the PDC is issued for individuals with mild to severe impairment, individuals with mild impairment might disproportionately be uncaptured by the WG-SS. For verifying this hypothesis, this study aimed to (1) examine the relationship between the severity of impairment and WG-SS judgment, and (2) describe percentages of individuals captured as “not having disability” based on the WG-SS stratified by severity of impairment among individuals who had the PDC of vision, hearing, or lower-limb impairments.

Methods: Secondary dataset of a representative Japanese individuals with disability (n = 14,079) was used for this cross-sectional study. From the dataset, data of individuals who had PDC of vision, hearing, or lower-limb impairments were extracted for analysis. All statistical analyses were conducted distinctively by the impairment group. After examining the relationship between severity of impairments and WG-SS judgement using univariate and multivariate analysis, percentages of individuals captured as “not having disability” based on a recommended cut point of the WG-SS, a response of “a lot of difficulty” or “cannot do at all” on at least one of the six questions, were calculated and described stratified by severity of impairment.

Results: The number of eligible study participants for analysis was as follows: PDC holders for vision impairment (n = 189), hearing impairment (n = 240), and lower-limb impairment (n = 634). The univariate or multivariate analysis showed significant relationships between the severity of impairment (mild impairment) and the difficulty category of corresponding WG-SS individual questions (mild difficulty, i.e., “no difficulty” or “some difficulty”) for each group. Among the PDC holders of vision impairments, the percentages of individuals captured as “not having disability” based on the WG-SS were 16.7% and 5.9% in the mild (n = 36) and severe (n = 153) impairment subgroups, respectively. Similarly, 39.0% and 20.2% in the mild (n = 146) and severe (n = 94) hearing impairment subgroups, respectively, and 54.0% and 28.9% in the mild (n = 430) and severe (n = 204) lower-limb impairment subgroups, respectively.

Conclusion: PDC holders with mild impairment were less likely to be captured by the WG-SS as “having disability” than those with severe impairment. These findings suggested a characteristic of the WG-SS that the disability statistics based on the WG-SS may underrepresent PDC holders with mild impairment. Stakeholders in Japan need to take into account this characteristic when they use or interpret the WG-SS.

## Introduction

The Washington Group Short Set on Functioning (WG-SS) is an international disability measure, aiming to estimate disability prevalence and disability-related inequalities globally [1]. As the WG-SS was initially developed to be incorporated in censuses or surveys, the measure is short and easy to administer. It has only six questions on functional difficulties such as seeing, hearing, and walking, with four answer options: no difficulty, some difficulty, a lot of difficulty, and cannot do at all [1]. For international comparison, disability status is defined by a cut point recommended by the WG-SS developers [1]. Specifically, a response of “a lot of difficulty” or “cannot do at all” to at least one of the six questions is defined as having disability. Since its adaptation in 2006, approximately 100 countries have incorporated the WG-SS in national censuses or surveys [2].

As the WG-SS is a relatively new disability measure, having better knowledge of the characteristics of the measure is essential for researchers and policymakers to appropriately interpret and use the data along with local and global contexts. In particular, an understanding of differences in how the new and existing disability measures capture people with disabilities is crucial because disability statistics are subject to change based on the choice of disability measure [3-6]. Apparent change in disability statistics induced by disability measure switching may lead to some kind of confusion as well as dispute among stakeholders [3,7-11]. Therefore, good knowledge of the differences is important to minimize the risks of these issues.

In 2022, the WG-SS was adopted for two nationwide surveys in Japan [12,13]. One of them is a representative survey of individuals with disabilities, i.e., Seikatsu no Shizurasa Chosa, which is translated as the Comprehensive Survey of Persons with Difficulties (CSPD) [14]. The adaptation is expected to enable Japanese stakeholders to review current Japanese disability-related policies based on internationally comparable disability statistics. However, caution is needed because exploration of the characteristics of the new measure is still in its infancy in Japan. Considering that possession of a physical disability certificate (PDC) has been recognized and used as a classic indicator of disability in Japan [15], information on compatibility between the two indicators is important for gaining better knowledge on characteristics of the WG-SS in the Japanese population.

A previous study in Japan [16] documented significant differences between disability prevalence based on the WG-SS and the existing disability indicator, i.e., possession of the PDC. Saito reported that approximately half of the persons who had PDC were not identified by the WG-SS as having disability [16]. This finding is consistent with previous studies in the US reporting disagreement between the WG-SS and existing disability indicators among people with vision, hearing, or mobility impairment [9,10]. According to the previous studies [9,10], among people with blind, deaf, or moderate and severe mobility disabilities who were identified by existing self-report-based disability indicators, approximately 30-50% of them were not captured by corresponding individual WG-SS questions as having severe difficulty per the WG-SS developer group’s suggested cut points.

As researchers of previous studies argued [9,10], exploring causes of the inconsistency is crucial for better understanding the WG-SS and for improving disability statistics. Considering that the PDCs are issued for individuals ranging from mild to severe impairment, those with less severe impairment might be disproportionately uncaptured by the WG-SS. This may be a possible explanation for the reported inconsistency in the previous study [16]. Unfortunately, however, this assumption was not examined in the previous study [16] and remains unclear. Bridging the knowledge gap would contribute to a better understanding of WG-SS’s unique characteristics in Japanese populations.

This study aimed to explore the characteristics of the WG-SS in the Japanese population. We hypothesized that PDC holders with less severe impairment were less likely to be captured by the WG-SS than those with severe one. To verify this hypothesis, we focused on Japanese PDC holders with vision, hearing, and lower-limb impairments, whose disability characteristics were comparable with study participants in the previous studies [9,10]. The similarity of the study samples’ characteristics would allow us to discuss current study findings in comparison to those of the previous studies [9,10]. First, we examined whether less severe impairment of vision, hearing, and lower limbs is a predictor of less severe difficulty category on corresponding individual WG-SS questions. Second, after stratifying the study participants by severity of impairment, we calculated and described percentages of study participants who were captured within each WG-SS difficulty category of the corresponding individual WG-SS question and were defined as being disabled using the suggested WG-SS cut point [1].

## Materials and methods

A cross-sectional study using secondary data of the CSPD was conducted. The authors obtained the secondary data from the Ministry of Health, Labour, and Welfare of Japan (MHLW) with permission to use the data for scientific purposes. The statistical data presented in the current study were computed by the authors. Therefore, the data may show some differences from officially published data by the MHLW.

This study was conducted based on the approval of the Ethics Committee of the National Rehabilitation Center for Persons With Disabilities (Approval No.: 2024-069). The informed consent procedure was waived due to the nature of the current study, i.e., secondary data analysis.

Overview of the CSPD

The CSPD has been conducted every five years since 2011 as a nationwide representative survey exclusively focusing on Japanese persons with disabilities. In 2022, the CSPD was conducted between December 1st and 22nd, which was the latest survey as of the current study was conducted [14].

A random sampling technique was used for obtaining a representative study sample of persons with disabilities in Japan [14]. Adults and children with any kind or form of difficulties in life due to physical, mental, or psychological issues were eligible for the CSPD. Individuals with PDC were one part of the eligible study samples. From approximately 5400 randomly selected stratified census tracts across Japan, eligible study participants were enumerated through every house-to-house visit by trained survey staff in the designated census tracts.

A self-administered questionnaire was provided to eligible study participants [17]. The eligible study participants were asked to fill out the questionnaire by themselves and return it by mail. When experiencing some difficulties in filling out the form, the eligible study participants were allowed to use a proxy response. Special accommodation, such as a questionnaire in Braille and sign language interpreters, was provided for the study participants upon request. Unfortunately, however, no detailed statistics on requested and provided special accommodation exist, making it difficult to estimate how the special accommodation needs were satisfied in the CSPD.

During the enumeration process, a total of 24,427 individuals with difficulty were recruited as eligible study participants and provided the questionnaire. Of these, 14,631 study participants filled out and returned the survey forms; the response rate was 59.9% [14]. Data from 552 individuals were judged as invalid and removed from the dataset by the MHLW. Consequently, a dataset consisting of 14,079 participants was provided to the authors as the secondary data of the CSPD.

Overview of the physical disability certificate in Japan

The PDC is issued by local governments for individuals with permanent physical disability based on the Act for the Welfare of Persons with Physical Disabilities (Act No. 283 of 1949). The PDC entitles holders to use designated medical, welfare, and rehabilitation services, and other support services, including financial support services [18].

A medical certificate and a written medical opinion by a designated medical doctor are necessary for applying the PDC [19]. These documents include information on medical diagnosis, types of impairment, and severity of impairment, according to the severity criteria stipulated by the Act. Subsequently, based on the medical information and other relevant information, the local welfare office or local government judges the applicant’s condition and issues the PDC, which carries information on the name of impairments and their severity. The PDC is issued primarily based on the diagnosis and other medical information provided by a designated medical doctor.

The PDC covers five types of permanent impairment: visual, hearing and equilibrium, speech, mobility, and visceral impairment [18]. Each impairment has zero to seven subcategories of impairment. For instance, mobility impairment has five subcategories: upper-limb impairment, lower-limb impairment, trunk impairment, upper-limb impairment induced by brain function disorder, and mobility impairment induced by brain function disorder. Consequently, in total, there are five types of impairment with 16 subcategories that PDC covers [18].

Severity of impairment in the PDC is divided into two to seven grades depending on the subcategory of impairment [18]. The smaller number of the severity grade indicates the more severe impairment. Table 1 shows the severity grades of specific subcategories on which the current study is focusing, i.e., vision, hearing, and lower-limb impairments. Vision impairment has six severity grades: grades 1 to 6. Similarly, hearing and lower-limb impairment have four and seven grades, respectively.

**Table 1:** Criteria for physical disability certificate of visual, hearing, and lower-limb impairments. NOTE: Original criteria, in Japanese, are available on the website of the Ministry of Health, Labour, and Welfare of Japan [18]. The translation from Japanese to English was conducted by the authors, consulting with designated medical doctors in rehabilitation medicine, otolaryngology, or ophthalmology.

	Visual impairment	Hearing impairment	Lower-limb impairment
Grade 1	Corrected visual acuity in better eye ≤ 0.01	NA	“Complete functional loss of both lower limbs” OR “Deficiency of both lower limbs proximal to mid-thigh”
Grade 2	“0.02 ≤ corrected visual acuity in better eye ≤ 0.03” OR “0.04 ≤ corrected visual acuity in better eye, and fellow eye ≤ hand motion” OR “Peripheral visual angle (I/4 isopter) was ≤ 80 degrees in each eye, and binocular central visual angle (I/2 isopter) was ≤ 28 degrees“ OR “≤70 binocular open-eye detectable points and ≤ 20 detectable points in the binocular central visual field”	≥100 dBHL in each ear	“Severe impairment of both lower limbs” OR “Deficiency of both lower limbs proximal to mid-leg”
Grade 3	“0.04 ≤ corrected visual acuity in better eye ≤ 0.07” OR “0.08 ≤ corrected visual acuity in better eye, and ≤ hand motion in fellow eye” OR “Peripheral visual angle (I/4 isopter) was ≤ 80 degrees in each eye, and binocular central visual angle (I/2 isopter) was ≤ 56 degrees” OR “≤ 70 binocular open-eye detectable points and ≤40 detectable points in the binocular central visual field”	≥90 dBHL in each ear	“Deficiency of both lower limbs proximal to Chopart joints” OR “Deficiency of one lower limb proximal to midthigh” OR “Complete functional loss of one lower limb”
Grade 4	“0.08 ≤ corrected visual acuity in better eye≤ 0.1” OR “Peripheral visual angle (I/4 isopter) was ≤ 80 degrees in each eye“ OR “≤70 binocular open-eye detectable points”	“≥80 dBHL in each ear” OR “≤50% of the best speech recognition threshold in each ear”	“Deficiency of all toes in both lower limbs” OR “Complete functional loss of all toes in both lower limbs” OR “Deficiency of one lower limb proximal to mid-leg” OR “Severe impairment of one lower limb” OR “Complete functional loss of hip or knee joint in one lower limb” OR “One lower limb is shortened as compared with the unaffected side by more than 10 cm or more than 1/10 of the unaffected side”
Grade 5	“0.2 ≤ corrected visual acuity in better eye, and ≤0.02 in fellow eye” OR “Visual field defect ≥ 50% in both eyes” OR “Binocular central visual angle (I/2 isopter) ≤ 56 degrees“ OR “70 ≤ binocular open-eye detectable points ≤ 100” OR “Peripheral visual angle (I/4 isopter) ≤ 80 degrees in each eye“ OR “Binocular open-eye detectable points ≤ 40”	NA	“Severe impairment of hip or knee joint in one lower limb” OR “Complete functional loss of ankle joint in one lower limb” OR “One lower limb is shortened as compared with the unaffected side by more than 5cm or more than 1/15 of the unaffected side”
Grade 6	0.3 ≤ corrected visual acuity in better eye ≤ 0.6, and visual acuity in fellow eye≤ 0.02	“≥70 dBHL in each ear” OR “≥90 dBHL in one ear, and ≥70 dBHL in fellow ear”	“Deficiency of one lower limb proximal to the Lisfranc joint” OR “Severe impairment of the ankle joint in one lower limb”
Grade 7	NA	NA	“Severe impairment of all toes in both lower limbs” OR “Mild impairment of one lower limb” OR “Mild impairment of hip, knee or ankle joint in one lower limb” OR “Deficiency of all toes in one lower limb” OR “Complete functional loss of all toes in one lower limb” OR “One lower limb is shortened as compared with the unaffected side by more than 3 cm or more than 1/20 of the unaffected side”

Inclusion and exclusion criteria for analysis

Inclusion criteria were set as individuals having PDC of vision, hearing, or lower-limb impairments aged five years and older because WG-SS was not recommended to apply to children under the age of five [1]. We excluded data of individuals who (a) had other types of disability-related certificates, i.e., mental disorder certificates and intellectual disability certificates, (b) had two or more types of impairments that were covered by the PDC, and (c) had missing values on variables for analysis, which will be described later. The intention of the exclusion criteria was to ensure that only PDC holders with vision, hearing, and lower-limb impairments who had no multiple impairments or disabilities were included for analysis.

Measures

Variables relating to demographic information (age and gender), proxy response, the PDC, and the WG-SS were used for the analysis.

Specific question on proxy response was “Who answered the questions?” Answer options were: “study participant him/herself,” “proxy who confirms answers of study participant, who was able to communicate with the proxy but not able to fill out answers by him/herself,” and “proxy who infers answers of study participant, who was not able to communicate with the proxy.”

There were two kinds of variables regarding the PDC in the CSPD: severity grade (grades 1 to 7) and age of issuance (age < 18, 18 <= age < 40, 40 <= age < 65, 65 <= age). The two variables were used for the analysis. The severity grade for each type of impairment was dichotomized as “severe impairment” and “mild impairment” using the midpoint: “Grade 1 to Grade 3” as severe impairment and ”Grade 4 to Grade 6” as mild impairment for the PDC of vision impairment, in the same manner, “Grade 2 and Grade 3” and ”Grade 4 and Grade 6” for the PDC of hearing impairment, and “Grade 1 to Grade 3” and ” Grade 4 to Grade 7” for the PDC of lower-limb impairment. As age of 65 years old is commonly used in Japan as a cut point to distinguish adults and older people, age of issuance was dichotomized as “age < 65” and “age >= 65.”

The specific six questions of the WG-SS are as follows: (1) Do you have difficulty seeing, even if wearing glasses? (2) Do you have difficulty hearing, even if using a hearing aid? (3) Do you have difficulty walking or climbing steps? (4) Do you have difficulty remembering or concentrating? (5) Do you have difficulty with self-care (such as washing all over or dressing)? (6) Using your usual language, do you have difficulty communicating (for example, understanding or being understood by others)? [1]. Answer options for the six questions are: “no difficulty,” “some difficulty,” “a lot of difficulty,” or “cannot do at all.” Difficulty category for individuals WG-SS questions was dichotomized based on the suggested cut point for international comparison [1]: “no difficulty” or “some difficulty” as “mild difficulty” and “a lot of difficulty” or “cannot do at all” as “severe difficulty.” Similarly, disability status based on the WG-SS was defined using the cut point; a response of “a lot of difficulty” or “cannot do at all” to at least one of the six questions is defined as having disability. Other answer patterns were defined as “not having disability.”

If an individual did not provide an answer for all six questions, the disability status was considered as a missing value. Moreover, if an individual did not provide an answer for some questions but answered the other questions as “no difficulty” or “some difficulty,” his/her disability status was also judged as a missing value because disability status is dependent on the true answers, that is unknown, for the questions with no answer.

Statistical analysis

As the study participants were distinct from each other based on their impairment types, they were divided into three groups: vision, hearing, and lower-limb impairment groups. Subsequently, all statistical analyses were conducted by the group. IBM SPSS Statistics for Windows version 28 (IBM Corp., Armonk, NY) was used for all statistical analyses. Statistical significance was defined as p < 0.05.

First, a descriptive analysis was conducted to present the study participants’ characteristics. Second, we compared the relationship between the severity of impairment and difficulty category on corresponding individual WG-SS questions using univariate and multivariate analysis. Specifically, after dividing study participants into two groups (i.e., mild and severe difficulty groups) based on the answer for the corresponding individual WG-SS, the chi-square test was used to analyze differences in demographic information, proxy response, and the PDC relating information between the two groups. Subsequently, binomial logistic regression analyses using the forced entry method were conducted to examine whether the severity of impairment was relevant to the WG-SS difficulty category after adjusting for confounding factors. Specifically, the severity of impairment, “mild impairment,” was the independent variable, whereas the WG-SS difficulty category, “mild difficulty,” was the dependent variable in the analysis model. Age, gender, proxy response, and age of PDC issuance were incorporated into the model as confounding factors. Model fit was evaluated using the model chi-square test. Lastly, we calculated and described percentages of the study participants who were captured within each WG-SS difficulty category and were defined as having disability by severity of impairment.

## Results

Participant selection process

**Figure 1:**
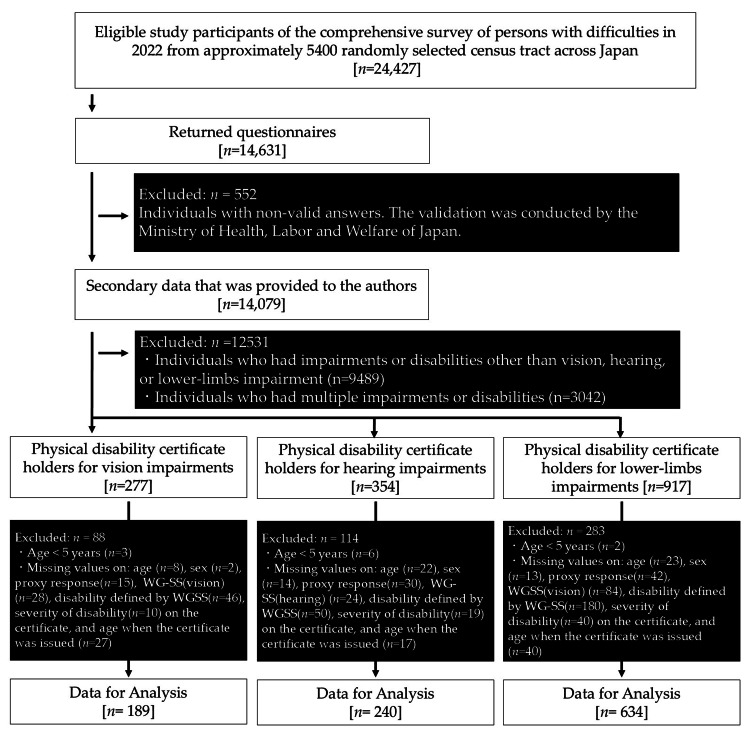
Participant selection process. WG-SS: Washington Group Short Set on Functioning.

Figure 1 shows the participant selection process. From a validated secondary dataset of 14,079 individuals, data of 277 to 917 individuals were extracted after removing data of individuals who had impairments or disabilities other than vision, hearing, or lower limbs, or who had multiple impairments or disabilities. After removing individuals who met the exclusion criteria, consequently, the eligible individuals for analysis were obtained: the PDC holders for vision impairment (n = 189), hearing impairment (n = 240), and lower-limb impairment (n = 634).

Study participants’ characteristics

Table 2 presents the characteristics of eligible study participants by WG-SS difficulty category. Approximately three-quarters of the study participants were aged 65 years or older in each three groups. Percentages of participants with “severe impairment” varied depending on the group: 81.0% (n = 153) for vision, 39.2% (n = 94) for hearing, and 32.2% (n = 204) for the lower-limb impairment.

**Table 2:** Characteristics of the study participants by WG-SS difficulty categories. The statistical parameters are presented in numbers and %. ^1^ Do you have difficulty seeing, even if wearing glasses? ^2^ Do you have difficulty hearing, even if using a hearing aid? ^3^ Do you have difficulty walking or climbing steps? ^†^ “A lot of difficulty” or “cannot do.” ^††^ “Some difficulty” or “no difficulty.” ^‡^ Proxy who confirms answers of the study participant, who was able to communicate with the proxy but not able to fill out the answers by him/herself. ^‡‡^ Proxy who infers answers of the study participant, who was not able to communicate with the proxy. ^§^ Grade 1 to grade 3 for physical disability certificate of vision impairment; grade 2 and grade 3 for physical disability certificate of hearing impairment; and grade 1 to grade 3 for physical disability certificate of lower-limb impairment. ^§§ ^Grade 4 to grade 6 for physical disability certificate of vision impairment; grade 4 and grade 6 for physical disability certificate of hearing impairment; and grade 4 to grade 7 for physical disability certificate of lower-limb impairment. WG-SS: Washington Group Short Set on Functioning.

		Physical disability certificate holders for vision impairment (n = 189)			Physical disability certificate holders for hearing impairment (n = 240)			Physical disability certificate holders for lower-limb impairment (n = 634)		
		Answer for corresponding WG-SS vision difficulty question^1^			Answer for corresponding WG-SS hearing difficulty question^2^			Answer for corresponding WG-SS walking difficulty question^3^		
		Severe^†^ (n = 166)	Mild^††^ (n = 23)	Total (n = 189)	Severe^†^ (n = 130)	Mild^†† ^(n = 110)	Total (n = 240)	Severe^†^ (n = 321)	Mild^†† ^(n = 313)	Total (n = 634)
Proxy response										
Self	Number	28	10	38	81	62	143	192	274	466
	(Column %)	(16.9)	(43.5)	(20.1)	(62.3)	(56.4)	(59.6)	(59.8)	(87.5)	(73.5)
Proxy 1^‡^	Number	104	9	113	23	24	47	74	21	95
	(Column %)	(62.7)	(39.1)	(59.8)	(17.7)	(21.8)	(19.6)	(23.1)	(6.7)	(15.0)
Proxy 2^‡‡^	Number	34	4	38	26	24	50	55	18	73
	(Column %)	(20.5)	(17.4)	(20.1)	(20.0)	(21.8)	(20.8)	(17.1)	(5.8)	(11.5)
Age										
5 ≤ Age < 65	Number	37	7	44	35	27	62	49	78	127
	(Column %)	(22.3)	(30.4)	(23.3)	(26.9)	(24.6)	(25.8)	(15.3)	(24.9)	(20.0)
Age ≥ 65	Number	129	16	145	95	83	178	272	235	507
	(Column %)	(77.7)	(69.6)	(76.7)	(73.1)	(75.5)	(74.2)	(84.7)	(75.1)	(80.0)
Gender										
Male	Number	92	15	107	64	50	114	111	111	222
	(Column %)	(55.4)	(65.2)	(56.6)	(49.2)	(45.5)	(47.5)	(34.6)	(35.5)	(35.0)
Female	Number	74	8	82	66	60	126	210	202	412
	(Column %)	(44.6)	(34.8)	(43.4)	(50.8)	(54.6)	(52.5)	(65.4)	(64.5)	(65.0)
Severity of impairment										
Severe^§^	Number	139	14	153	65	29	94	134	70	204
	(Column %)	(83.7)	(60.9)	(81.0)	(50.0)	(26.4)	(39.2)	(41.7)	(22.4)	(32.2)
Mild^§§^	Number	27	9	36	65	81	146	187	243	430
	(Column %)	(16.3)	(39.1)	(19.1)	(50.0)	(73.6)	(60.8)	(58.3)	(77.6)	(67.8)
Age of issue										
5 ≤ Age < 65	Number	95	14	109	74	44	118	171	246	417
	(Column %)	(57.2)	(60.9)	(57.7)	(56.9)	(40.0)	(49.2)	(53.3)	(78.6)	(65.8)
Age ≥ 65	Number	71	9	80	56	66	122	150	67	217
	(Column %)	(42.8)	(39.1)	(42.3)	(43.1)	(60.0)	(50.8)	(46.7)	(21.4)	(34.2)

Relationship between the severity of impairment and difficulty category on the corresponding individual WG-SS questions

Table 3 shows the results of comparison analysis using the chi-square test between the mild and severe difficulty groups based on the answer to the corresponding individual WG-SS question. In all three impairment groups, the mild difficulty group showed significantly higher percentages of mild impairment: 16.3% (n = 27) in severe difficulty group and 39.1% (n = 9) in mild difficulty group among the PDC holders of vision impairment (p = 0.019); similarly, 50.0% (n = 65) in severe difficulty and 73.6% (n = 81) in mild difficulty among the PDC holders for hearing impairment (p =< 0.001), and 58.3% (n = 187) in severe difficulty and 77.6% (n = 243) in mild difficulty among the PDC holders for lower-limb impairment (p =< 0.001).

**Table 3:** Comparison of the characteristics between WG-SS difficulty categories. The statistical parameters are presented in numbers and %. ^1^ Do you have difficulty seeing, even if wearing glasses? ^2^ Do you have difficulty hearing, even if using a hearing aid? ^3^ Do you have difficulty walking or climbing steps? ^*^ Chi-square test. ^†^ “A lot of difficulty” or “cannot do.” ^††^ “Some difficulty” or “no difficulty.” ^‡^ Proxy who confirms answers of the study participant, who was able to communicate with the proxy but not able to fill out the answers by him/herself. ^‡‡^ Proxy who infers answers of the study participant, who was not able to communicate with the proxy. ^§^ Grade 1 to grade 3 for physical disability certificate of vision impairment; grade 2 and grade 3 for physical disability certificate of hearing impairment; and grade 1 to grade 3 for physical disability certificate of lower-limb impairment. ^§§^ Grade 4 to grade 6 for physical disability certificate of vision impairment; grade 4 and grade 6 for physical disability certificate of hearing impairment; and grade 4 to grade 7 for physical disability certificate of lower-limb impairment. WG-SS: Washington Group Short Set on Functioning.

		Physical disability certificate holders for vision impairment (n = 189)			Physical disability certificate holders for hearing impairment (n = 240)			Physical disability certificate holders for lower-limb impairment (n = 634)		
		Answer for corresponding WG-SS vision difficulty question^1^			Answer for corresponding WG-SS hearing difficulty question^2^			Answer for corresponding WG-SS walking difficulty question^3^		
		Severe^†^ (n = 166)	Mild^††^ (n = 23)	P-value^* ^(Chi-square value)	Severe^†^ (n = 130)	Mild^†† ^(n = 110)	P-value^* ^(Chi-square value)	Severe^†^ (n = 321)	Mild^†† ^(n = 313)	P-value^* ^(Chi-square value)
Proxy										
Self	Number	28	10	0.018 (9.080)	81	62	0.617 (0.966)	192	274	<0.001 (62.660)
	(Column %)	(16.9)	(43.5)		(62.3)	(56.4)		(59.8)	(87.5)	
Proxy 1^‡^	Number	104	9		23	24		74	21	
	(Column %)	(62.7)	(39.1)		(17.7)	(21.8)		(23.1)	(6.7)	
Proxy 2^‡‡^	Number	34	4		26	24		55	18	
	(Column %)	(20.5)	(17.4)		(20.0)	(21.8)		(17.1)	(5.8)	
Age										
5 ≤ age < 65	Number	37	7	0.386 (0.750)	35	27	0.675 (0.176)	49	78	0.002 (9.223)
	(Column %)	(22.3)	(30.4)		(26.9)	(24.6)		(15.3)	(24.9)	
Age ≥ 65	Number	129	16		95	83		272	235	
	(Column %)	(77.7)	(69.6)		(73.1)	(75.5)		(84.7)	(75.1)	
Gender										
Male	Number	92	15	0.374 (0.789)	64	50	0.559 (0.341)	111	111	0.816 (0.054)
	(Column %)	(55.4)	(65.2)		(49.2)	(45.5)		(34.6)	(35.5)	
Female	Number	74	8		66	60		210	202	
	(Column %)	(44.6)	(34.8)		(50.8)	(54.6)		(65.4)	(64.5)	
Severity of impairment										
Severe^§^	Number	139	14	0.019 (6.850)	65	29	<0.001 (13.971)	134	70	<0.001 (27.275)
	(Column %)	(83.7)	(60.9)		(50.0)	(26.4)		(41.7)	(22.4)	
Mild^§§^	Number	27	9		65	81		187	243	
	(Column %)	(16.3)	(39.1)		(50.0)	(73.6)		(58.3)	(77.6)	
Age of issue										
5 ≤ age < 65	Number	95	14	0.741 (0.110)	74	44	0.009 (6.828)	171	246	<0.001 (45.142)
	(Column %)	(57.2)	(60.9)		(56.9)	(40.0)		(53.3)	(78.6)	
Age ≥ 65	Number	71	9		56	66		150	67	
	(Column %)	(42.8)	(39.1)		(43.1)	(60.0)		(46.7)	(21.4)	

Table 4 shows the results of the binomial logistic regression analysis. The model chi-square test indicated that overall predictive models with all independent variables were statistically significant, except for the PDC holders for vision impairment (p = 0.055). Therefore, adjusted odds ratios (ORs) for the PDC holders for hearing and lower-limb impairment and unadjusted ORs for the PDC holders for vision impairment are described in Table 4. After adjusting for confounding factors, “mild impairment” was found to be a significant predictor for “mild difficulty” on the corresponding individual WG-SS questions (adjusted OR (95% CI): 2.482 (1.346-4.578) among the PDC holders for hearing impairment and 2.415 (1.664-3.505) among the PDC holders for hearing impairment). Unadjusted OR (95% CI) for the PDC holders for vision impairment was 3.310 (1.301-8.416).

**Table 4:** Unadjusted and adjusted odds ratios of the severity of vision, hearing, and lower-limb impairments for the WG-SS difficulty category, “mild difficulty.” The statistical parameters are presented in odds ratios. Statistical significance was defined as p < 0.05. ^1^ Do you have difficulty seeing, even if wearing glasses? ^2^ Do you have difficulty hearing, even if using a hearing aid? ^3^ Do you have difficulty walking or climbing steps? ^†^ “Some difficulty” or “no difficulty.” ^††^ Confounding factors adjusted: age, gender, proxy response, and age of disability certificate issue. ^‡^ Grade 1 to grade 3 for physical disability certificate of vision impairment; grade 2 and grade 3 for physical disability certificate of hearing impairment; and grade 1 to grade 3 for physical disability certificate of lower-limb impairment. ^‡‡^ Grade 4 to grade 6 for physical disability certificate of vision impairment; grade 4 and grade 6 for physical disability certificate of hearing impairment; and grade 4 to grade 7 for physical disability certificate of lower-limb impairment. NA: not applicable; WG-SS: Washington Group Short Set on Functioning; PDC: physical disability certificate.

	Answer for corresponding WG-SS vision difficulty question^1^as“mild difficulty^†^” in PDC holders for vision impairment		Answer for corresponding WG-SS hearing difficulty question^2 ^as “mild difficulty^†^” in PDC holders for hearing impairment		Answer for corresponding WG-SS walking difficulty question^3 ^as “mild difficulty^†^” in PDC holders for lower-limb impairment	
	Unadjusted OR (95% CI)	Adjusted OR^††^ (95% CI)	Unadjusted OR (95% CI)	Adjusted OR^††^ (95% CI)	Unadjusted OR (95% CI)	Adjusted OR^††^ (95% CI)
Model chi-square test	P = 0.016	P = 0.055	P < 0.001	P = 0.006	P < 0.001	P < 0.001
With severe impairments^‡^	Ref.	NA	Ref.	Ref.	Ref.	Ref.
With mild impairments^‡‡^	3.310 (1.301–8.416)	NA	2.793 (1.618–4.822)	2.482 (1.346–4.578)	2.488 (1.760–3.517)	2.415 (1.664–3.505)

Percentage of physical disability certificate holders who were captured within each WG-SS difficulty category and were defined as being disabled using WG-SS

**Figure 2:**
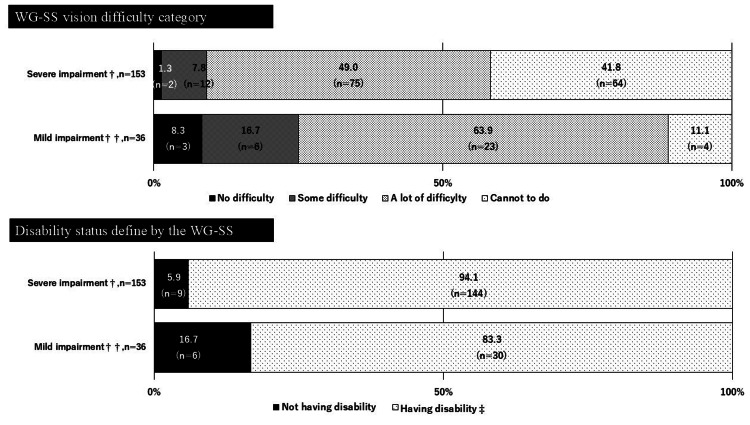
Percentage of physical disability certificate holders for vision impairments who were captured within each WG-SS vision difficulty category and were defined as being disabled using the suggested WG-SS cut point. The statistical parameters are presented in numbers and %. ^†^ Grade 1 to grade 3 for the physical disability certificate of vision impairment. ^††^ Grade 4 to grade 6 for the physical disability certificate of vision impairment. ^‡^ Response of “a lot of difficulty” or “cannot do at all” to at least one of the six WG-SS questions is defined as having disability. WG-SS: Washington Group Short Set on Functioning.

**Figure 3:**
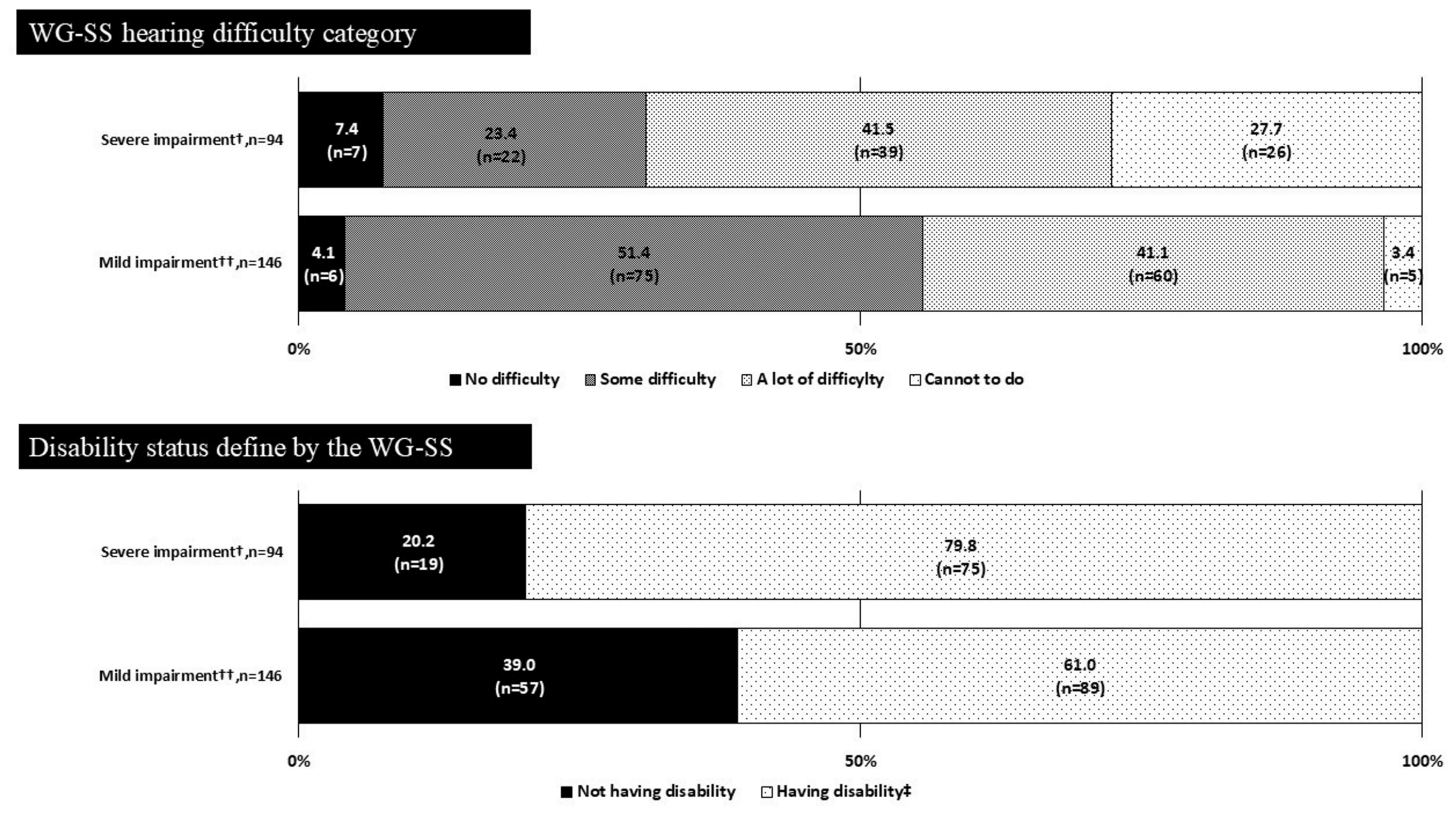
Percentage of physical disability certificate holders for hearing impairments who were captured within each WG-SS hearing difficulty category and were defined as being disabled using the suggested WG-SS cut point. The statistical parameters are presented in numbers and %. ^†^ Grade 2 and grade 3 for the physical disability certificate of hearing impairment. ^††^ Grade 4 and grade 6 for the physical disability certificate of hearing impairment. ^‡^ Response of “a lot of difficulty” or “cannot do at all” to at least one of the six WG-SS questions is defined as having disability. WG-SS: Washington Group Short Set on Functioning.

**Figure 4:**
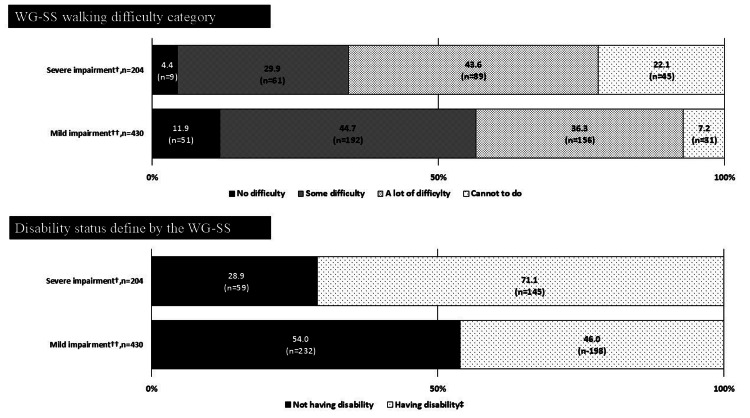
Percentage of physical disability certificate holders for lower-limb impairments who were captured within each WG-SS walking difficulty category and were defined as being disabled using the suggested WG-SS cut point. The statistical parameters are presented in numbers and %. ^†^ Grade 1 to grade 3 for the physical disability certificate of lower-limb impairments. ^††^ Grade 4 to grade 7 for the physical disability certificate of lower-limb impairments. ^‡^ Response of “a lot of difficulty” or “cannot do at all” to at least one of the six WG-SS questions is defined as having disability. WG-SS: Washington Group Short Set on Functioning.

Figures 2-4 illustrate the percentage of PDC holders who were captured within each WG-SS difficulty category and were defined as having disability by severity of impairment. Figure 2 showed that, among the PDC holders of vision impairment with mild impairment (n = 36), 8.3% (n = 3) and 16.7% (n = 6) were captured as “no difficulty” and “some difficulty” for the corresponding vision question on the WG-SS, respectively. These figures were relatively higher than those with severe vision impairment (n = 153), 1.3% (n = 2) for “no difficulty” and 7.8% (n = 12) for “some difficulty,” respectively. Subsequently, the percentage of having no disability in those with mild impairment (16.7%, n = 6) was 2.84 times higher than those with severe impairment (5.9%, n = 9). Similar gaps between the PDC holders with mild and severe impairment were observed among those with hearing and lower-limb impairment (Figures 3, 4); percentages of having no disability in those with mild impairment were 1.93 times and 1.87 times higher than those with severe impairment among the PDC holders of hearing and lower-limb impairment, respectively.

## Discussion

Our univariate and multivariate analyses showed that “mild impairment” was a significant predictor for “mild difficulty” on the corresponding individual WG-SS question among PDC holders for vision, hearing, and lower-limb impairments. Consequently, the PDC holders with mild impairment were less likely to be captured by the WG-SS as having disability than those with severe impairment. These findings suggested a WG-SS’s characteristic that disability statistics based on the WG-SS may underrepresent PDC holders with mild impairment. Stakeholders in Japan need to take into account this characteristic when they use or interpret the WG-SS.

Performance of the WG-SS in capturing the PDC holders with mild vision, hearing, and lower-limb impairments

As we hypothesized, the PDC holders with mild impairment were disproportionately uncaptured by the WG-SS than those with severe impairment. The percentages of PDC holders with mild impairment who were identified as “not having disability” by the WG-SS were approximately two to three times higher than those with severe impairment. This finding is consistent with a study [3] reporting the WG-SS’s characteristics that the WG-SS is likely to capture more vulnerable people, i.e., those who had multiple disabilities, and less likely to capture less vulnerable people, i.e., those who had a single type of disability.

One plausible reason for the findings is the difference in the way of assessing disability status. Specifically, the WG-SS is primally based on subjective difficulty from respondents, whereas the PDC is primally issued based on medical diagnosis by medical doctors. It is not surprising that gaps exist between subjective difficulty and medical diagnosis, i.e., a person with mild impairment who meets the criteria for the PDC does not necessarily feel difficulties in his/her life.

Although compatibility between the WG-SS and existing self-report-based disability indicator has been well documented in previous studies [3-6], little is known about compatibility between the WG-SS and medical diagnosis-based disability indicator, an alternative, objectively-assessed disability benchmark. The current study suggested that there may be a certain degree of discordance between them, especially among those with mild impairments. Although this knowledge was yielded from a study focusing on the Japanese population and its generalizability to other geological contexts was not examined, our findings could serve as a base for further research exploring characteristics of the WG-SS in other nations' contexts.

Difference in the WG-SS’s performance between impairment types

Overall, the PDC holders of hearing or lower-limb impairments had higher percentages of “mild difficulty” for the corresponding individual WG-SS question and “not having disability” defined by the WG-SS than those of vision impairment. The PDC holders of hearing or lower-limb impairments showed wider gaps between subjective difficulty and medical diagnosis than their counterparts. Our findings are consistent with the previous studies in the US reporting relatively higher percentages of “no difficulty” or “some difficulty” for corresponding individual WG-SS questions among people with hearing [10] or severe mobility disabilities [9] than those with vision disabilities [9].

Although the exact reasons for this finding are unclear, a possible explanation is that the impact of assistive devices or adaptive equipment on daily life may vary depending on impairment type. For instance, even if a person had severe hearing or lower-limb impairment, assistive devices or adaptive equipment, such as a cochlear implant [20] and artificial limbs [21,22], may, in part, compensate his/her impairment, enabling him/her to live with minor difficulties. Assistive devices or adaptive equipment for visual impairments, such as magnifying glasses or closed-circuit television, would also be helpful. However, their impact on daily life may not necessarily be sufficient for people with vision impairment to live with minor difficulty.

The WG-SS was intended to be developed for capturing difficulties in basic activities in a non-accommodating environment [23]. Inclusion of assistive devices was restricted only to seeing and hearing questions in the WG-SS, as these difficulties can often be easily overcome with the use of glasses or hearing aids in most countries [24]. For instance, question specifications of the WG-SS [24] say that “the capacity to walk should be without assistance of any device (wheelchair, crutches, walker, etc.) or human.” Therefore, responses to the WG-SS are supposed to be immune to the influence of assistive devices or adaptive equipment. However, the CSPD was conducted as a self-administered questionnaire survey, which did not include the aforementioned explanation on the use of assistive devices for answering the WG-SS questions. This unique survey procedure in the CSPD may also have contributed to widening the gap between subjective difficulty and medical diagnosis in the PDC holders of hearing or lower-limb impairments.

Potential alternative cut point of the WG-SS for the Japanese population

For defining the disability status, the current study used a cut point that was recommended by the WG-SS developers for international comparison [1]. However, as the WG-SS developers stated, an appropriate cut point needed to be dictated by the outcome of interest and the need for the data [1].

For assuring comparability of disability statistics across countries, it would be reasonable to use the recommended cut point in the global context. While from a Japanese domestic viewpoint, an alternative cut point of the WG-SS that can harmonize better with the existing disability indicator in Japan, the PDC, may be more useful.

A possible alternative cut point, the authors would like to suggest based on the current study findings, is “some difficulty in at least one of six questions of the WG-SS.” If the wider cut point were applied in the current study, approximately 90% of the PDC holders would be captured in the corresponding individual WG-SS question. Consequently, the wider cut point would likely provide disability statistics that harmonize better with the PDC. Previous studies [3-6] supported this assumption that wider cut points yield disability statistics that were more consistent with existing disability statistics. Hanass-Hancock and colleagues recommended including “some difficulty” for identifying disability status as a possible alternative cut point [25]. Further studies are necessary to explore characteristics of the WG-SS using different cut points in the local Japanese context.

Limitations and strengths

Several study limitations need to be considered when interpreting the current study findings. First, this study focused on the PDC holders of vision, hearing, or lower-limb impairments in Japan and excluded PDC holders of other types of impairment or multiple impairments. Therefore, the results of the current study are only applicable to the specific populations. Generalizability of the study findings to PDC holders with other types of impairment in Japan or individuals in other countries is not assured. Second, the response rate of the CSPD was not satisfactory (59.9%). Arguably, the non-respondents might have represented more vulnerable individuals. The incomplete responses might have introduced selection bias and underestimated the gap between the PDC holders with mild and severe impairment. Third, as noted above, further research exploring alternative cut points, which are more suitable in the local Japanese context, is necessary for a better understanding of the characteristics of the WG-SS.

## Conclusions

The PDC holders with mild impairment were more likely to be identified by the WG-SS as “not having disability” than those with severe impairment, with two to three times higher percentages of identification among those with mild impairment. These findings suggested a characteristic of the WG-SS that the disability statistics based on the WG-SS may underrepresent PDC holders with mild impairment. Stakeholders in Japan need to take into account this characteristic when they use or interpret the WG-SS.
